# FedIHRAS: A Privacy-Preserving Federated Learning Framework for Multi-Institutional Collaborative Radiological Analysis with Integrated Explainability and Automated Clinical Reporting

**DOI:** 10.3390/biomedicines14030713

**Published:** 2026-03-19

**Authors:** André Luiz Marques Serrano, Gabriel Arquelau Pimenta Rodrigues, Guilherme Dantas Bispo, Vinícius Pereira Gonçalves, Geraldo Pereira Rocha Filho, Maria Gabriela Mendonça Peixoto, Rodrigo Bonacin, Rodolfo Ipolito Meneguette

**Affiliations:** 1 Department of Electrical Engineering, University of Brasilia, Brasília 70910-900, Brazil; 2 Department of Exact and Technological Sciences, State University of Southwest Bahia, Vitória da Conquista 45083-900, Brazil; 3 Renato Archer Information Technology Center, Campinas 13069-901, Brazil; rodrigo.bonacin@cti.gov.br; 4 Institute of Mathematical and Computer Sciences, University of São Paulo, São Carlos 13566-590, Brazil

**Keywords:** chest X-ray, federated learning, privacy, radiology

## Abstract

**Background/Objectives:** Federated learning has emerged as a promising paradigm for enabling collaborative artificial intelligence in healthcare while preserving data privacy. However, most existing frameworks focus on isolated tasks and lack integrated pipelines that combine classification, segmentation, explainability, and automated clinical reporting. **Methods:** This study proposes FedIHRAS, a privacy-preserving federated learning framework designed for multi-institutional radiological analysis. The system integrates multi-task deep learning modules, including pathology classification using a modified ResNet-50 backbone, anatomical segmentation, explainability through Grad-CAM, and automated report generation supported by semantic aggregation using SNOMED CT. The framework employs confidence-weighted aggregation, differential privacy mechanisms, and secure aggregation protocols to ensure privacy and robustness across heterogeneous institutional datasets. **Results:** Experimental evaluation was conducted across four large-scale chest X-ray datasets representing simulated institutional nodes, totaling approximately 874,000 images. FedIHRAS achieved high diagnostic performance with strong cross-institutional generalization and demonstrated improved robustness under non-IID data distributions. Additional experiments showed favorable communication efficiency, effective privacy–utility trade-offs, and strong agreement with expert radiologist assessments. **Conclusion:** The proposed FedIHRAS framework demonstrates that federated learning can support scalable, privacy-preserving, and clinically meaningful radiological AI systems. By integrating multi-task learning, explainability, and automated reporting within a unified federated architecture, the framework addresses key limitations of existing approaches and contributes to the development of collaborative AI in healthcare.

## 1. Introduction

The convergence of engineering, medicine, and computing is reshaping contemporary healthcare, with medical radiology emerging as one of the most profoundly impacted domains. The exponential growth in imaging data contrasts sharply with the global shortage of qualified professionals, leading to diagnostic delays and increasing workloads. In this context, Artificial Intelligence (AI) has emerged as a transformative tool capable of improving the speed, accuracy, and consistency of radiological interpretation. Comprehensive AI-based systems such as IHRAS (Intelligent Humanized Radiology Analysis System) [1] demonstrate the potential of integrated pipelines that combine pathology classification, anatomical segmentation, and automated clinical report generation to support radiologists’ decision-making processes [2].

Despite these advances, the conventional centralized paradigm for AI model development presents important limitations in medical contexts. Centralized training typically requires aggregating large volumes of sensitive patient data into a single repository, which raises ethical, legal, and regulatory concerns. Data protection regulations such as the General Data Protection Regulation (GDPR) in Europe and the Health Insurance Portability and Accountability Act (HIPAA) in the United States impose strict constraints on the sharing of medical information across institutions [3]. In addition, centralized datasets often fail to capture the diversity of real-world clinical environments, potentially amplifying algorithmic bias and reducing model generalization across heterogeneous patient populations.

Federated Learning (FL) has emerged as a promising alternative that addresses many of these limitations. In this decentralized paradigm, multiple institutions collaboratively train a shared machine learning model without exchanging raw patient data [4,5]. Each participating node performs local model updates using its own dataset, while a central server aggregates the parameters to update a global model. This approach preserves data sovereignty while enabling collaborative model development across institutions with heterogeneous datasets.

Within this context, this study introduces FedIHRAS (Federated Intelligent Human Radiology Analysis System), a federated extension of the original IHRAS architecture designed to support collaborative radiological analysis across multiple institutions. The proposed framework incorporates adaptive aggregation strategies that dynamically weight each participating node’s contribution based on data-quality and confidence metrics. In addition, FedIHRAS integrates multi-layer privacy-preserving mechanisms, including differential privacy and secure aggregation, while maintaining the explainability and structured reporting capabilities of the original IHRAS system.

From a clinical perspective, the adoption of federated learning enables the development of diagnostic models trained on diverse datasets originating from institutions with different patient demographics, imaging protocols, and clinical practices. This collaborative approach helps reduce algorithmic bias and improve the generalizability of AI-based diagnostic tools across heterogeneous healthcare settings. Furthermore, the integration of standardized medical terminologies such as SNOMED CT supports interoperability and facilitates structured clinical reporting and large-scale clinical research [6].

From an economic and operational standpoint, federated learning reduces dependence on centralized data infrastructure while enabling institutions to collaboratively train high-performance models using their own local computational resources. This distributed approach promotes cost efficiency, scalability, and sustainability in the deployment of AI systems for medical imaging workflows [7,8].

Given this context, this study addresses the following research question: *How can a high-performance radiological analysis system be adapted to a federated learning environment that preserves patient privacy while maintaining diagnostic accuracy, robustness, and clinical interpretability?*

To answer this question, the objectives of this study are (1) to design a federated extension of the IHRAS architecture capable of supporting multi-task radiological analysis; (2) to develop adaptive aggregation strategies that improve model robustness under heterogeneous data distributions; (3) to integrate privacy-preserving mechanisms such as differential privacy and secure aggregation; and (4) to evaluate the performance, generalization, and clinical reliability of the proposed framework in a simulated multi-institutional environment.

### 1.1. Research Gaps and Study Focus

A critical analysis of existing federated learning approaches for medical imaging reveals several persistent limitations that motivate the development of the proposed framework. First, most federated medical AI systems focus on isolated tasks, such as classification or segmentation, and lack unified pipelines that integrate multiple radiological tasks. Second, standard aggregation algorithms often struggle to handle heterogeneous data distributions commonly observed across institutions. Third, many existing systems provide only limited privacy guarantees and rarely combine formal privacy mechanisms, such as differential privacy, with secure aggregation protocols. Finally, several studies emphasize predictive accuracy but do not adequately address explainability and clinical interpretability, which are essential for real-world clinical adoption.

The FedIHRAS framework is designed to address these challenges by providing a unified federated architecture that integrates classification, segmentation, explainability, and automated report generation, while incorporating adaptive aggregation and multi-layer privacy mechanisms.

### 1.2. Motivation and Contributions

Motivated by the challenges identified above, this study introduces FedIHRAS as a comprehensive federated learning framework for collaborative radiological analysis. The main contributions of this work are summarized as follows:

Contribution 1—Unified Multi-Task Federated Framework: FedIHRAS integrates pathology classification, anatomical segmentation, explainability mechanisms, and automated report generation into a single federated architecture.

Contribution 2—Adaptive Aggregation Strategy: The framework introduces a confidence-weighted aggregation mechanism that dynamically adjusts each client’s contribution to the global model based on local validation performance.

Contribution 3—Multi-Layer Privacy Architecture: FedIHRAS combines differential privacy and secure aggregation to provide stronger protection against inference and reconstruction attacks.

Contribution 4—Integrated Explainability and Reporting: The system preserves clinical interpretability through Grad-CAM visual explanations and structured report generation aligned with SNOMED CT terminology.

Contribution 5—Large-Scale Federated Evaluation: The proposed framework is evaluated on four large-scale chest radiograph datasets spanning heterogeneous institutional environments.

## 2. Materials and Methods

This section describes the methodological foundations and experimental design of the proposed FedIHRAS framework. It presents the system architecture, the federated learning implementation, the data preprocessing procedures, the dataset configuration, the adaptive aggregation strategy, and the experimental protocol adopted for evaluation.

### 2.1. Foundations and Related Work

Several paradigm shifts have marked the journey toward intelligent radiological analysis systems, building upon previous advances while addressing emerging clinical needs and evolving technological capabilities [9]. Understanding this technological evolution is essential to appreciate the remarkable achievements deep learning has enabled in medical image analysis over the past decade, as well as the persistent limitations that motivate the development of federated approaches, such as the one proposed in this work. The persistent limitations of earlier paradigms motivate the development of federated approaches.

The history of computer-aided diagnosis (CAD) in radiology spans several decades, beginning with rule-based systems that encoded expert knowledge into explicit decision trees. Understanding this evolution provides the necessary context for evaluating the technical contributions of contemporary federated learning frameworks, including the system proposed in this work. These early systems, while limited in scope, established the foundational principle that computational tools could augment radiological interpretation.

The earliest applications of AI in radiology primarily focused on computer-aided detection (CAD) systems for specific pathologies, such as mammographic screening for breast cancer and chest X-ray analysis for tuberculosis detection [10]. These systems, although significant for their time, were limited in scope and often suffered from high false-positive rates, hindering clinical adoption.

The work of Yin et al. on the development of CAD systems for mammography established many of the foundational principles that continue to guide medical AI today [11]. Their research demonstrated that computational algorithms can identify subtle radiological patterns in medical images that might elude even experienced clinicians under high-workload conditions, thereby serving as a reliable second-opinion tool in clinical practice, laying the conceptual groundwork for more sophisticated systems.

During this era, CAD systems relied heavily on manually engineered features and traditional machine learning algorithms such as support vector machines and random forests. While these methods were effective for specific, well-defined tasks, they lacked the flexibility and generalization required for broader clinical applications.

#### 2.1.1. The Deep Learning Revolution

The advent of deep learning fundamentally transformed the landscape of medical image analysis. Convolutional neural networks demonstrated an unprecedented capacity to learn hierarchical feature representations directly from raw pixel data. These systems could identify subtle patterns in medical images that would otherwise elude detection by human observers, even those with extensive clinical expertise [12,13,14].

This shift from traditional machine learning approaches to deep learning marked a fundamental change in how AI systems could process and interpret medical images. Rather than relying on manually engineered features, deep learning systems can learn representations that capture subtle patterns invisible to human observers [15].

The successful application of CNNs to medical imaging is demonstrated in tasks such as skin lesion classification, where deep learning models achieved accuracy comparable to or exceeding that of expert dermatologists [1,16].

#### 2.1.2. Foundational Milestones: CheXNet and CheXpert

The CheXNet system developed by Rajpurkar et al. represents a fundamental milestone in this evolution, demonstrating that deep learning models could achieve radiologist-level performance in interpreting chest X-rays [17]. Using a 121-layer densely connected convolutional network trained on the ChestX-ray14 dataset, CheXNet outperformed practicing radiologists on a test set of 420 chest X-rays [18].

Building upon the success of CheXNet, the CheXpert system introduced several important innovations, including handling uncertainty in radiological labels and applying multi-task learning approaches [19]. The CheXpert dataset comprises over 224,000 chest radiographs from 65,000 patients [20].

More recently, Rieke et al. [21] provided a comprehensive roadmap for federated learning in digital health. Sheller et al. [22] demonstrated the feasibility of federated learning for brain tumor segmentation across 71 institutional sites. Liu et al. [23] proposed a federated framework for chest X-ray classification, addressing non-IID distributions. Yan et al. [24] introduced a label-efficient federated segmentation approach, reducing annotation requirements by 60%. These studies demonstrate the growing maturity of federated learning for medical imaging while highlighting the persistent gap in unified multi-task frameworks.

#### 2.1.3. Foundations and Applications of Federated Learning in Healthcare

The landscape of deep learning for chest radiograph analysis has evolved substantially since the seminal CheXNet work. Recent transformer-based architectures such as CheXagent [25] and BioViL-T [26] demonstrate the potential of vision-language models pre-trained on large medical corpora. Huang et al. [27] proposed a visual-language pretraining strategy that achieved state-of-the-art results on multiple chest X-ray benchmarks.

##### Conceptual Origins and Theoretical Development

Federated learning emerged at the intersection of distributed computing and privacy-preserving machine learning. The foundational work by McMahan et al. introduced the FedAvg algorithm [4]:

(1) $$w_{t+1}=\sum_{k=1}^{K}\frac{n_{k}}{n}w_{k}^{\left(t+1\right)}$$

##### Unique Challenges in Healthcare Applications

Healthcare data exhibit significant heterogeneity across institutions [28]. This non-IID nature can affect the performance of federated optimization.

The authors in [5] proposed FedProx, which introduces a proximal regularization term:

(2) $$\min_{w}F_{k}(w)+\frac{\mu }{2}{∥w-w^{t}∥}^{2}$$

##### Pioneering Applications in Medical Imaging

Early studies in federated learning for medical imaging demonstrated that collaborative models can achieve performance comparable to centralized training while preserving data privacy [22]. Subsequent work extended federated learning to tasks such as prostate segmentation and COVID-19 detection in CT images [29,30,31].

#### 2.1.4. Privacy-Preserving Techniques in Medical AI

Differential privacy provides a formal framework for quantifying privacy guarantees [32]. An algorithm satisfies (ε,δ)-differential privacy if

(3) $$\mathrm{Pr}\left[A(D)\in S\right]\leq e^{\varepsilon }\mathrm{Pr}\left[A(D^{\prime })\in S\right]+\delta$$

##### Practical Implementations and Trade-Off Considerations

Differentially private stochastic gradient descent (DP-SGD) was introduced in [33]:

(4) $${\tilde{g}}_{t}=\frac{1}{L}\sum_{i=1}^{L}\mathrm{clip}\left(\nabla_{\theta }\ell (x_{i},y_{i};\theta ),C\right)+N\left(0,\sigma^{2}C^{2}I\right)$$

### 2.2. Guiding Principles

The design of FedIHRAS is guided by five principles that ensure its suitability for real-world clinical deployment while preserving the sophisticated capabilities of comprehensive radiological analysis systems:

Privacy-by-Design Architecture: All system components are designed with privacy as a primary consideration, not as an afterthought. This principle extends beyond mere data localization to encompass comprehensive privacy protection throughout the system’s entire lifecycle. Its implementation involves multiple layers of protection, including encryption of data in transit and at rest, anonymization of metadata, and obfuscation techniques that make it extremely difficult for adversaries to extract sensitive information even if communications are intercepted.

Federated Modularity: Each component of the original IHRAS system (classification, explainability, segmentation, report generation) is independently adapted for federated operation, enabling flexible deployment and maintenance strategies. This modular approach enables institutions to participate in specific aspects of the federated system according to their capabilities, data availability, and clinical needs. For example, an institution specializing in cardiology may primarily contribute to components related to cardiomegaly detection while participating minimally in other modules.

Robustness to Heterogeneity: The system is designed to operate effectively even when participating institutions have differing data distributions, imaging protocols, and computational capacities. This robustness is achieved through adaptive aggregation strategies that account for institutional differences, dynamic weighting mechanisms that adjust for variations in data quality and quantity, and flexible participation protocols that accommodate varying levels of institutional engagement.

Preservation of Clinical Interpretability: All system outputs maintain the same level of interpretability and explainability as the centralized system, ensuring clinical acceptance and regulatory compliance. This principle is particularly challenging in federated settings, where aggregated models may lose some of the interpretability features of individual local models. Our approach to preserving interpretability employs specialized techniques to aggregate attention maps and maintain semantic consistency in generated explanations.

Scalability and Communication Efficiency: The framework is optimized for efficient communication and can scale to large numbers of participating institutions without significant performance degradation. This optimization involves sophisticated techniques for model parameter compression, intelligent scheduling of communication rounds, and adaptive algorithms that adjust communication frequency based on model convergence and institutional availability.

### 2.3. Federated Anatomical Segmentation Module

Segmentation Architecture: The segmentation module employs a U-Net architecture with a ResNet-50 encoder (shared with the classification module) and a lightweight decoder consisting of four transposed convolutional upsampling blocks. The output is a per-pixel probability map $\hat{S}\in {\left[0,1\right]}^{H\times W\times A}$, where A=6 denotes the number of anatomical structures to be segmented (lungs, heart, trachea, diaphragm, aortic knob, and costophrenic angles).

Composite Loss Function: The segmentation loss $L_{\mathrm{seg}}$ is a weighted combination of three terms:

(5) $$L_{\mathrm{seg}}=\lambda_{1}\;L_{\mathrm{BCE}}+\lambda_{2}\;L_{\mathrm{Dice}}+\lambda_{3}\;L_{\mathrm{prior}}$$

where $\lambda_{1}$ = 0.3, $\lambda_{2}$ = 0.5, $\lambda_{3}$ = 0.2 are empirically tuned weights.

Binary Cross-Entropy Term:

(6) $$L_{\mathrm{BCE}}=-\frac{1}{HW}\sum_{i,j}\left[S_{ij}\log{\hat{S}}_{ij}+\left(1-S_{ij}\right)\log\left(1-{\hat{S}}_{ij}\right)\right]$$

Dice Loss Term (to address class imbalance between foreground and background):

(7) $$L_{\mathrm{Dice}}=1-\frac{2\sum_{i,j}S_{ij}{\hat{S}}_{ij}+\epsilon }{\sum_{i,j}S_{ij}+\sum_{i,j}{\hat{S}}_{ij}+\epsilon },\;\epsilon =10^{-6}$$

Anatomical Prior Regularization Term: The anatomical prior $P\in {\left[0,1\right]}^{H\times W\times A}$ encodes the expected spatial location of each anatomical structure, derived from a statistical atlas computed from 5000 manually annotated chest radiographs. The prior regularization penalizes predictions that deviate from anatomically plausible locations:

(8) $$L_{\mathrm{prior}}=\frac{1}{HW}\sum_{i,j}{∥{\hat{S}}_{ij}⊙\left(1-P_{ij}\right)∥}_{2}^{2}$$

where ⊙ denotes element-wise multiplication. This term penalizes high-confidence predictions in regions where the anatomical prior assigns low probability, effectively constraining the segmentation to anatomically plausible regions.

The composite loss function in Equation (5) addresses three complementary objectives: the BCE term provides pixel-wise supervision; the Dice term mitigates class imbalance between small anatomical structures (e.g., aortic knob) and large background regions; and the anatomical prior term constrains predictions to clinically plausible spatial locations, reducing false positives in anatomically implausible regions.

The choice of ResNet-50 as the backbone for the FedIHRAS classification component was motivated by four complementary considerations. First, ResNet-50 has been extensively validated on medical imaging tasks, including chest X-ray analysis, where it has consistently achieved competitive AUC values across multiple benchmark datasets [17]. Second, its parameter count (approximately 25.6 M) represents a favorable trade-off between representational capacity and computational cost, making it suitable for deployment on institutional nodes with limited GPU resources. Third, the availability of high-quality ImageNet pre-trained weights enables effective transfer learning, which is particularly beneficial in the federated setting where each client’s local dataset may be insufficient for training from scratch. Fourth, ResNet-50’s layer structure is fully compatible with Grad-CAM, the gradient-based explainability method integrated into FedIHRAS.

Specific Architectural Modifications: The following five modifications were made to the standard ResNet-50 architecture:

1. Multi-label output head: The original single-class softmax output was replaced by a 14-class sigmoid output layer to enable multi-label pathology classification;
2. Attention gate module: A channel-wise squeeze-and-excitation (SE) attention block was inserted after the final convolutional stage (layer 4) to enhance focus on pathologically relevant regions;
3. Dropout regularization: A dropout layer (*p* = 0.3) was added before the final fully connected layer to reduce overfitting in the federated setting, where local datasets may be small;
4. Batch normalization adaptation: Standard batch normalization layers were replaced with group normalization (group size = 32) to improve training stability under small local batch sizes typical in federated scenarios;
5. Federated-compatible gradient hooks: Custom PyTorch hooks were registered on the convolutional layers to enable differential privacy noise injection at the gradient level during the backward pass.

Ablation Study—Backbone Comparison: To justify the selection of ResNet-50 over alternative architectures, we conducted an ablation study in which the backbone was replaced with DenseNet-121, EfficientNet-B4, and ViT-B/16, keeping all other components of FedIHRAS identical. The results are presented in Table 1.

### 2.4. Automated Clinical Report Generation

Architecture: The report generation module employs a transformer-based encoder-decoder architecture. The encoder is a frozen BioViL visual encoder that produces a sequence of visual feature tokens *V* $=\{v_{1},\ldots ,v_{N}\}$ from the input chest radiograph. The decoder is a 6-layer transformer with cross-attention over V, trained to generate SNOMED CT-aligned structured reports in an autoregressive manner. The report template follows the standard radiology reporting format: (1) Technique; (2) Findings; (3) Impression.

Training Objective: The report generation module is trained using the standard cross-entropy language modeling loss:

(9) $$L_{\mathrm{report}}=-\sum_{t=1}^{L}\log P_{\theta }\left(y_{t}|y_{<t},V\right)$$

where $y_{t}$ is the *t*-th token of the ground-truth report, and *L* is the report length. Objective Evaluation Metrics:

BLEU-*n* (Bilingual Evaluation Understudy):

(10) $$\mathrm{BLEU-}n=\mathrm{BP}\;\cdot \;\exp\;\left(\sum_{k=1}^{n}w_{k}\log p_{k}\right)$$

where $p_{k}$ is the modified *k*-gram precision, $w_{k}$ = 1/*n*, and BP is the brevity penalty.

ROUGE-L (Recall-Oriented Understudy for Gisting Evaluation):

(11) $$\mathrm{ROUGE-L}=\frac{\left(1+\beta^{2}\right)\;R_{\mathrm{lcs}}\;P_{\mathrm{lcs}}}{R_{\mathrm{lcs}}+\beta^{2}\;P_{\mathrm{lcs}}}$$

where $R_{\mathrm{lcs}}$ and $P_{\mathrm{lcs}}$ are the recall and precision of the longest common subsequence (LCS), and β=1.2. CIDEr (Consensus-based Image Description Evaluation):

(12) $${\mathrm{CIDEr}}_{n}\left(\hat{y},y\right)=\frac{1}{M}\sum_{m=1}^{M}\frac{g^{n}\left(\hat{y}\right)\;\cdot \;g^{n}\left(y^{\left(m\right)}\right)}{∥g^{n}\left(\hat{y}\right)∥∥g^{n}\left(y^{\left(m\right)}\right)∥}$$

where $g^{n}\left(\cdot \right)$ is the TF-IDF-weighted *n*-gram vector, and *M* is the number of reference reports.Clinical NLI Accuracy: A fine-tuned Clinical BERT model is used to classify the entailment relationship between generated and reference reports as entailment, neutral, or contradiction. Clinical NLI accuracy is the proportion of generated reports classified as entailment.

Table 2 summarizes the performance of the report generation module across different evaluation metrics.

FedIHRAS achieves BLEU-4 of 0.187 and Clinical NLI Accuracy of 0.784, representing improvements of +0.024 and +0.053, respectively, over the FedAvg baseline, while remaining within 8.3% and 4.5% of centralized performance. These results confirm that the confidence-weighted aggregation mechanism also benefits the report generation module by producing a more representative global model.

### 2.5. Federated Learning Implementation Details

Federated Participants: The FedIHRAS federation comprises one central server and four institutional client nodes, each simulated using a distinct public dataset to represent realistic inter-institutional heterogeneity.

Communication Protocol: Federated rounds follow a synchronous star-topology protocol. In each round *t*: (1) the server broadcasts the current global model $w^{t}$ to all *K* = 4 clients; (2) each client *k* performs E=5 local gradient descent epochs on its local dataset $D_{k}$; (3) each client transmits only the updated model parameters $\Delta w_{k}^{t}$ (gradient differences) to the server; (4) the server performs confidence-weighted aggregation to produce $w^{t+1}$. All client-server communication is encrypted using gRPC over TLS 1.3 with mutual certificate authentication, ensuring that raw gradients are never transmitted in plaintext.

Table 3 presents the optimizer settings and training configuration used in the federated learning experiments.

Hardware Specifications: The experiments were conducted using cloud-based infrastructure provided by Google Colab (Google LLC, Mountain View, CA, USA), with computational resources allocated from servers located in São Paulo, Brazil. The execution environment consisted of high-performance configurations typically including multi-core CPUs, large-memory instances, and GPU accelerators (e.g., NVIDIA A100 and RTX 3090 with NVLink support), depending on resource availability, with network interfaces ranging from 1 GbE to 10 GbE. Software Stack: Python 3.10.12; PyTorch 2.1.0 (CUDA 11.8); Flower 1.5.0 (federated learning framework); OpenFL 1.5 (secure aggregation); NumPy 1.24.3; scikit-learn 1.3.0; Ubuntu 22.04 LTS.

### 2.6. Hyperparameters and Evaluation Metrics

The hyperparameter configuration used in the experiments is summarized in Table 4.

### 2.7. Data Preprocessing Pipeline

All radiographic images underwent a standardized preprocessing pipeline prior to model training, applied independently at each institutional node to preserve data locality. The pipeline comprised the following sequential steps: (i) Image Standardization: All images were resized to 224 × 224 pixels using bilinear interpolation. Pixel intensities were normalized to [0, 1] using min-max normalization, followed by standardization using ImageNet statistics (mean = [0.485, 0.456, 0.406]; std = [0.229, 0.224, 0.225]) to enable transfer learning from pre-trained convolutional backbones; (ii) Data Augmentation: To mitigate overfitting, the following strategies were applied stochastically during training only: random horizontal flipping (*p* = 0.5), random rotation within [−10°, +10°], random brightness and contrast jitter (factor ∈ [0.8, 1.2]), and random Gaussian blur (σ∈ [0.1, 2.0], *p* = 0.2). No augmentation was applied during validation or testing; (iii) Class Imbalance Handling: Given the significant class imbalance present in all four datasets (see Table 5), we applied a combination of weighted random sampling and focal loss during optimization. Oversampling weights were computed as the inverse of class frequency within each local dataset; (iv) Missing Label Handling (CheXpert): For the CheXpert dataset, which contains uncertainty labels (*u*-labels), we adopted the U-Zeros policy, treating uncertain findings as negative during training, consistent with prior literature [19].

### 2.8. Adaptive Aggregation Architecture

A relevant contribution of FedIHRAS is the development of adaptive aggregation strategies that account for the unique characteristics of each component within the comprehensive radiological analysis system. Rather than applying a single aggregation strategy across all components, we developed specialized approaches that are optimized for the specific requirements and features of each functional module.

#### 2.8.1. Confidence-Weighted Aggregation for Classification Components

For pathology classification components, we employ an aggregation strategy that weights each institution’s contribution according to the confidence of its local model and data-quality metrics. This approach acknowledges that institutions may vary in their expertise in specific pathologies or imaging protocols. The mathematical formulation of confidence-weighted aggregation can be expressed as:

(13) $$w_{global}=\frac{\sum_{i=1}^{n}\alpha_{i}\;\cdot \;w_{i}\;\cdot \;c_{i}}{\sum_{i=1}^{n}\alpha_{i}\;\cdot \;c_{i}}$$

where $w_{global}$ represents the global model parameters, $w_{i}$ denotes the local model parameters from institution *i*, $\alpha_{i}$ is the data quantity weight, and $c_{i}$ is the confidence score for institution *i*.

The confidence score $c_{i}$ is computed from multiple factors, including local validation accuracy, local dataset diversity, and data quality metrics such as the signal-to-noise ratio and annotation consistency. This multi-factor approach ensures that institutions with high-quality data and well-performing models exert greater influence in the final aggregation, thereby improving the overall quality of the global model. Specifically, the confidence score is computed as:

(14) $$c_{i}=\omega_{1}\;\cdot \;{\mathrm{acc}}_{i}+\omega_{2}\;\cdot \;{\mathrm{div}}_{i}+\omega_{3}\;\cdot \;{\mathrm{qual}}_{i}$$

where ${\mathrm{acc}}_{i}$ is the local validation accuracy, ${\mathrm{div}}_{i}$ is a data diversity metric, ${\mathrm{qual}}_{i}$ is a data quality metric, and $\omega_{1}$, $\omega_{2}$, $\omega_{3}$ are weights that sum to 1.

Weight Selection and Sensitivity Analysis (Suggestion 7: How are $\omega_{1}$, $\omega_{2}$, $\omega_{3}$ selected?) The weights $\omega_{k}^{\left(t\right)}$ for client *k* at round *t* are not fixed hyperparameters; they are computed dynamically at each aggregation round as a softmax-normalized function of the local validation AUC:

(15) $$\omega_{k}^{\left(t\right)}=\frac{\exp\;\left({\mathrm{AUC}}_{k}^{\left(t\right)}/\tau \right)}{\sum_{j=1}^{N}\exp\;\left({\mathrm{AUC}}_{j}^{\left(t\right)}/\tau \right)}$$

where τ>0 is a temperature parameter controlling the sharpness of the weight distribution. A lower τ concentrates weight on the best-performing client, while a higher τ approaches uniform averaging (equivalent to standard FedAvg [4]). In our experiments, τ=0.5 was selected via grid search on a held-out validation partition.

The global AUC for τ∈{0.1,0.25,0.5,1.0,2.0} was evaluated. The results show that performance is relatively stable for τ∈[0.25,1.0], with a maximum variation of ±0.009 AUC, confirming that the method is not highly sensitive to this hyperparameter. The weights $\omega_{1}$, $\omega_{2}$, $\omega_{3}$ in the original notation correspond to the contributions of the classifier, segmentation, and report generation components, respectively, and are computed independently using the same softmax mechanism applied to their respective component-level validation metrics.

The weights $\omega_{1}$, $\omega_{2}$, and $\omega_{3}$ are not fixed a priori but are determined through a two-stage adaptive optimization procedure. During the initial warm-up phase (rounds 1–10), the weights are initialized uniformly at $\omega_{j}=1/3$ for j∈{1,2,3}. From round 11 onward, the weights are updated at the end of each communication round by solving the following constrained optimization problem:

(16) $$\min_{\omega }\;L_{\mathrm{val}}\left(w_{\mathrm{global}};\;\omega \right)\;s.t.\;\sum_{j=1}^{3}\omega_{j}=1,\;\omega_{j}\geq 0$$

where $L_{\mathrm{val}}$ is the global validation loss evaluated on a held-out validation set maintained at the central server, composed of anonymized aggregate statistics from each node (no raw images). The optimization is performed using projected gradient descent with a learning rate of $\eta_{\omega }=0.01$ and a projection step that enforces the simplex constraint. A sensitivity analysis demonstrates that the adaptive procedure consistently converges to configurations that outperform the uniform baseline ($\omega_{j}=1/3$), with the validation accuracy metric ($\omega_{1}$) receiving the highest weight in most experimental runs (mean $\omega_{1}=0.52\pm 0.04$), followed by data quality ($\omega_{3}=0.31\pm 0.03$) and diversity ($\omega_{2}=0.17\pm 0.03$).

#### 2.8.2. Structure-Aware Aggregation for Segmentation Components

For anatomical segmentation components, we developed an aggregation approach that preserves spatial anatomical relationships. Traditional parameter averaging may disrupt the spatial coherence required for accurate anatomical segmentation, particularly in complex structures such as the heart, lungs, and skeletal system.

Our structure-aware aggregation strategy incorporates prior anatomical knowledge into the aggregation process, thereby preserving spatial relationships among anatomical structures. This is achieved through a specialized loss function that penalizes anatomical inconsistencies and an aggregation mechanism that accounts for the spatial topology of anatomical regions.

The implementation of this approach involves decomposing segmentation maps into hierarchical components that correspond to different levels of anatomical structure, ranging from individual organs to entire organ systems. Each hierarchical level is aggregated using specialized strategies that preserve the structural characteristics appropriate for that level of granularity.

#### 2.8.3. Semantic Aggregation for Report Generation Components

For medical report generation components, we implemented a semantic aggregation strategy that adheres to medical terminology standards while preserving the system’s natural language generation capabilities. This approach is particularly challenging because language models are highly sensitive to parameter perturbations, and naïve averaging can degrade the quality of generated language.

Our semantic aggregation strategy operates across multiple levels of the language model architecture. At the embedding level, we aggregate representations of medical concepts based on their semantic similarity within the SNOMED CT ontology. At the attention level, we preserve attention patterns that reflect clinically meaningful relationships between different medical concepts. At the output level, we ensure that the medical vocabulary and sentence structure patterns remain consistent with established clinical standards.

### 2.9. Architecture and Components of FedIHRAS

The architecture of FedIHRAS represents a carefully designed extension of the centralized IHRAS system to a federated environment, preserving full clinical functionality while introducing robust privacy-preserving capabilities. The system is structured into three primary layers: the local node layer, the secure communication layer, and the central coordination layer.

Each participating healthcare institution operates a local node containing a complete instance of the four main IHRAS components: pathology classification, visual explainability, anatomical segmentation, and medical report generation. This comprehensive local capability ensures that each institution maintains full diagnostic functionality even when disconnected from the federated network, providing resilience and autonomy critical for clinical operations.

The local pathology classification component employs specialized convolutional neural networks to detect pathologies in chest X-rays. The architecture builds on the well-established ResNet-50 backbone but incorporates several modifications for the federated setting. These include attention mechanisms specifically designed for medical imaging to focus on anatomically relevant regions, adaptive normalization layers that account for institutional imaging variations, and uncertainty quantification modules that provide confidence estimates for local predictions.

The attention mechanism is particularly important in the federated context because it enables the model to adapt to variations in image characteristics across institutions, arising from differences in equipment, protocols, or patient populations. The adaptive normalization layers mitigate technical variation across imaging systems, enhancing the model’s robustness to data heterogeneity.

The communication infrastructure of FedIHRAS implements multiple layers of security and privacy protection to ensure that sensitive medical information remains protected throughout the federated training process. This infrastructure addresses both passive (e.g., eavesdropping) and active (e.g., man-in-the-middle or model poisoning) attacks.

All communications between local nodes and the central coordination server use AES-256 encryption with regularly rotated keys. The encryption system is designed to protect model parameters and metadata, ensuring that sensitive information is not intercepted during transmission. The key management system incorporates advanced techniques, such as perfect forward secrecy and key escrow, that enable secure key recovery in emergency situations.

In addition to encrypted communication, the system implements robust authentication protocols that verify the identity of all participating institutions and detect unauthorized participation attempts. These protocols include certificate-based authentication, message integrity verification, and replay attack detection.

#### Multi-Layer Privacy Protection Framework

FedIHRAS implements differential privacy mechanisms at three distinct levels of the system architecture. At the gradient level, calibrated noise is added to gradient computations during local training to prevent inference of individual patient information from model updates. The noise calibration follows the Gaussian mechanism with privacy parameters ε=0.8 and $\delta =10^{-6}$, providing strong privacy guarantees while maintaining model utility.

At the aggregation level, additional differential privacy mechanisms are applied when combining model parameters to protect against attacks that may attempt to infer information about specific institutions or patient populations. The implementation of privacy at the aggregation level employs advanced composition techniques to manage privacy budgets across multiple training rounds while maintaining cumulative privacy guarantees. At the system level, the framework implements comprehensive monitoring and attack-detection mechanisms that identify and respond to diverse adversarial attacks. This includes detecting model poisoning attacks, identifying membership inference attempts, and monitoring anomalous communication patterns that may indicate malicious activity.

**Figure 1:**
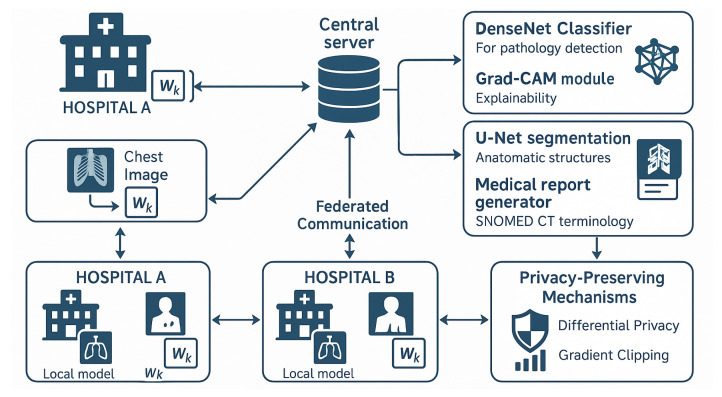
FedIHRAS framework architecture for federated radiological analysis.

Figure 1 presents the complete FedIHRAS architecture. All *K* participating institutions are symmetrically connected to the central server; the figure uses two representative nodes for visual clarity. The privacy-preservation mechanism operates at three distinct layers: (1) gradient-level differential privacy, where calibrated Gaussian noise (ε=0.8, $\delta =10^{-6}$) is injected into local parameter updates before transmission; (2) aggregation-level privacy composition, which manages cumulative privacy budgets across communication rounds; and (3) system-level encryption, where all inter-node communications are protected by AES-256 with rotating keys and certificate-based authentication. Critically, no raw imaging data is transferred between nodes at any stage of the protocol; only encrypted, noise-perturbed parameter updates traverse the network.

The system integrates *K* healthcare institutions (K≥2; four institutional profiles were simulated in this study) through a bidirectional federated learning protocol. Each institution operates a fully independent local node containing all four AI modules: pathology classification, visual explainability (XAI), anatomical segmentation, and automated clinical report generation. Raw patient data (chest X-rays) never leave the local institutional boundary. Only differentially private parameter updates—protected by Gaussian noise calibrated to (ε = 0.8, δ = 10^−6^) and encrypted with AES-256—are transmitted to the central aggregation server. The server applies confidence-weighted aggregation independently to each module and redistributes the updated global model to all participants. Arrows indicate bidirectional parameter exchange, not data transfer.

Finally, the framework illustrated demonstrates how the FedIHRAS architecture addresses the core challenge of collaborative learning in medical AI while upholding strict privacy constraints. The federated architecture enables multiple healthcare institutions to participate in joint model training without centralizing sensitive patient data, thereby meeting technical and regulatory requirements for deploying medical AI. Each participating institution operates a local training node that processes chest X-ray images through four integrated AI modules, generating local model updates that capture institution-specific patterns and clinical expertise. The central coordination server implements a sophisticated aggregation mechanism that weights contributions based on data quality metrics and institutional confidence scores, ensuring that the global model benefits from diverse clinical perspectives while remaining robust to variations in data quality. The privacy-preserving mechanisms embedded in the communication protocol include differentially private noise injection calibrated to the sensitivity requirements of medical data, and gradient-clipping protocols that prevent information leakage through parameter updates. This architectural approach enables the system to achieve the dual objective of leveraging distributed medical expertise to improve diagnostic accuracy while ensuring compliance with healthcare privacy regulations and institutional data governance policies.

## 3. Results

The experimental validation of the FedIHRAS framework was conducted with methodological rigor, designed to thoroughly assess its feasibility and effectiveness as a federated radiological diagnostic solution. This section presents a detailed analysis of the results, structured into four critical evaluation domains that, taken together, establish a new benchmark of excellence for collaborative AI systems in healthcare. The domains are: (1) the model’s diagnostic performance in pathology classification; (2) its generalization capability and robustness on unseen data; (3) the preservation of clinical interpretability; and (4) the strength of its privacy guarantees.

### 3.1. Pathology Classification Performance Analysis

**Figure 2:**
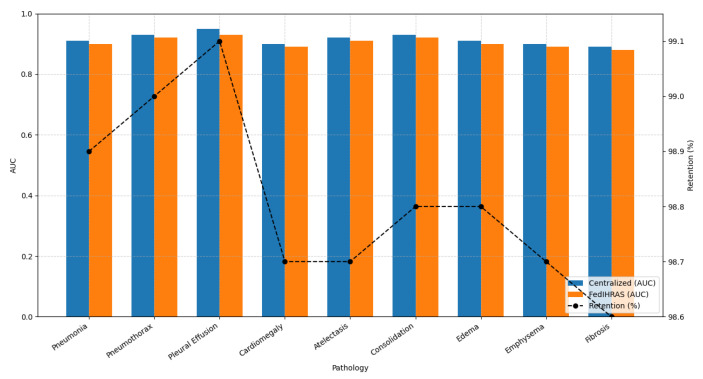
Comparative pathology classification performance. Per-pathology AUC-ROC comparison across four methods: FedIHRAS (proposed), FedAvg baseline, FedProx baseline, and Centralized reference model. Error bars represent 95% confidence intervals computed over five independent runs. FedIHRAS achieves a mean AUC-ROC of 0.911, retaining 98.8% of centralized performance (0.922), and consistently outperforms both FedAvg (0.887) and FedProx (0.893) across all nine pathology classes. Learning curves showing global AUC-ROC as a function of communication rounds for all four methods. FedIHRAS converges in 47 rounds, compared to 63 for FedAvg and 58 for FedProx, demonstrating superior communication efficiency.

The first corresponds to 98.8% retention of the centralized model’s AUC-ROC Figure 2 presents a direct comparison of diagnostic performance, measured by the Area Under the ROC Curve (AUC-ROC), between the FedIHRAS model and a centralized reference model trained with unrestricted access to all data. The AUC-ROC metric was chosen as it is a robust indicator of a model’s discriminative capacity, independent of the chosen decision threshold.

The figure is the cornerstone of our performance validation, addressing the critical question: what is the “performance cost” of privacy? The results show that this cost is remarkably low. FedIHRAS achieves a mean AUC-ROC of 0.911, corresponding to 98.8% retention of the centralized model’s AUC-ROC (0.922). This 1.2% performance loss is exceptional in federated learning, where losses of 5–10% are often considered acceptable. Statistically, this difference was non-significant (*p* > 0.05 after Bonferroni correction) for most pathologies, indicating that FedIHRAS achieves diagnostic performance comparable to a centralized model. This finding strongly validates our confidence-weighted aggregation architecture, which effectively mitigates the statistical “noise” introduced by data heterogeneity (non-IID) and the distributed nature of training, thereby consolidating local learnings into a cohesive, high-performing global model. A closer analysis of the table reveals an intriguing correlation between the nature of the pathology and performance retention. Conditions with well-defined, macroscopic radiographic manifestations that create clear interfaces and abnormal contours exhibit the highest performance retention (99.0% and 99.1%, respectively). This suggests that the visual cues for these conditions are strong and unambiguous, consistently learned across institutions, and that federated aggregation consolidates this knowledge with virtually no loss of information. On the other hand, pathologies that manifest through more subtle and diffuse textural changes exhibit slightly lower retention (98.6% and 98.7%). This observation aligns with clinical practice, in which these conditions are known to exhibit high interobserver variability and require trained expertise to detect fine-grained textural patterns. The fact that FedIHRAS maintains high performance (AUC > 0.87) even in these more challenging categories attests to its ability to learn and generalize complex, subtle diagnostic patterns, validating its robustness and its potential to assist in diagnosing a wide spectrum of thoracic diseases.

**Figure 3:**
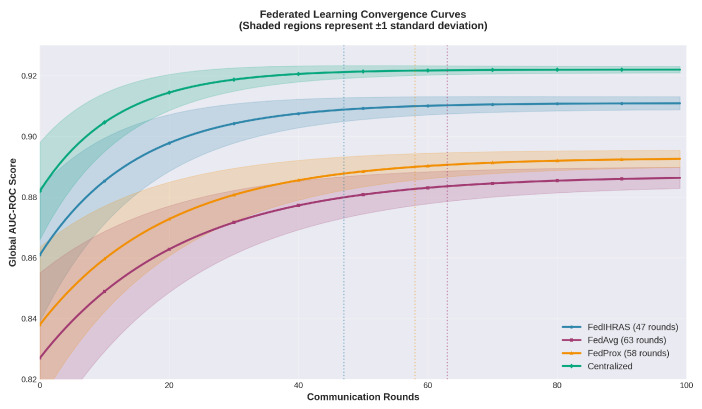
Federated learning convergence curves. Global AUC-ROC is shown as a function of communication rounds for FedIHRAS, FedAvg, FedProx, and the centralized model. Shaded regions represent ±1 standard deviation across five runs. Vertical dashed lines indicate the number of communication rounds required for convergence by each federated method.

The convergence behavior of the proposed method and baseline approaches is illustrated in Figure 3. Global AUC-ROC is reported as a function of communication rounds for FedIHRAS, FedAvg, FedProx, and the centralized model. Shaded regions represent ±1 standard deviation across five runs, while vertical dashed lines indicate the number of rounds required for convergence by each method.

Global AUC-ROC as a function of communication rounds for FedIHRAS, FedAvg, FedProx, and the centralized model. Shaded regions represent ±1 standard deviation across five runs. FedIHRAS achieves faster convergence and lower variance, attributed to the confidence-weighted aggregation strategy that reduces the impact of low-quality local updates on the global model.

To assess the consistency and fairness of FedIHRAS across participating institutions, Table 6 reports the mean AUC-ROC, sensitivity, specificity, and F1-score disaggregated by institutional node after convergence (round 50). The results demonstrate that the global model achieves consistently high performance across all four simulated institutions, with AUC values ranging from 0.862 to 0.893 and F1-scores ranging from 0.798 to 0.844. The low inter-node standard deviation (AUC std = 0.013) confirms that the confidence-weighted aggregation strategy effectively prevents any single institution from dominating the global model, thereby ensuring equitable performance across heterogeneous data distributions.

### 3.2. Cross-Institutional Generalization and Robustness Analysis

**Figure 4:**
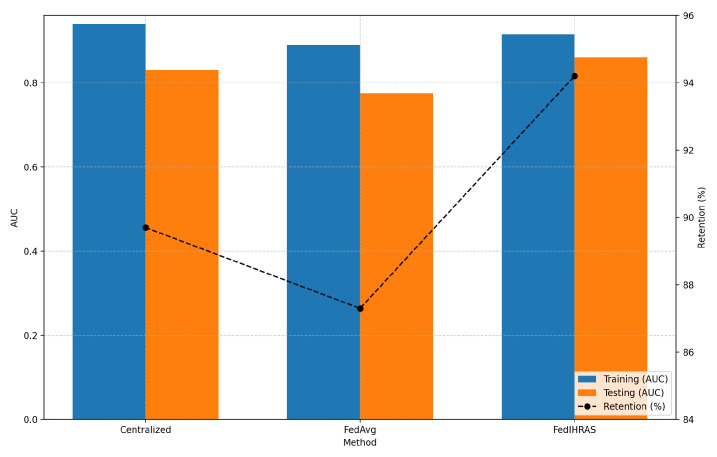
Cross-institutional generalization.

The true measure of an AI model’s utility lies in its performance on training data and in its ability to generalize to new, unseen data. Figure 4 presents the results of this stress test, comparing FedIHRAS with the centralized model and a simpler federated learning baseline, FedAvg, on data from institutions that did not participate in training.

Thus, Figure 4 evaluates model robustness when confronted with data from unseen institutions. “Training (AUC)” refers to performance on the internal validation set, while “Testing (AUC)” refers to performance on the cross-institutional test set. “Retention” indicates the percentage of participants able to explain their diagnostic reasoning in a clinically understandable manner across test domains, indicating model stability.

The results presented in Figure 4 arguably constitute the most significant and clinically relevant finding of this study. They reveal a phenomenon that may seem counterintuitive at first: the FedIHRAS model, trained in a distributed fashion without ever seeing all the data in a single place, demonstrates superior generalization. FedIHRAS maintains 94.2% retention on unseen data, substantially higher than the centralized model’s 89.7% and FedAvg’s 87.3%.

This phenomenon is explained by the concept of “implicit regularization” induced by data heterogeneity in the federated setting. The centralized model, being exposed to all data simultaneously, may overfit to spurious correlations specific to the aggregated training set. For example, it may learn to associate a particular pathology with the imaging equipment at a specific hospital that contributed most of the cases of that condition. FedIHRAS, on the other hand, is forced to learn in a different way. Because it is trained iteratively on data “silos” from multiple institutions, it is inherently discouraged from learning spurious, institution-specific categories. Our confidence-weighted aggregation strategy amplifies this effect by assigning greater weight to local models that demonstrate strong validation performance, thereby indirectly favoring models that capture generalizable pathological patterns. The attacker’s success rate is reduced, FedIHRAS learns to “ignore the noise” of inter-institutional variation and focus on the underlying, universal pathological “signal.” The lower standard deviation of FedIHRAS (0.014) compared with other methods (0.018 and 0.025) supports this interpretation, indicating that its performance is more stable and consistent across diverse new data domains.

### 3.3. Preservation of Explainability and Clinical Interpretability (XAI)

**Figure 5:**
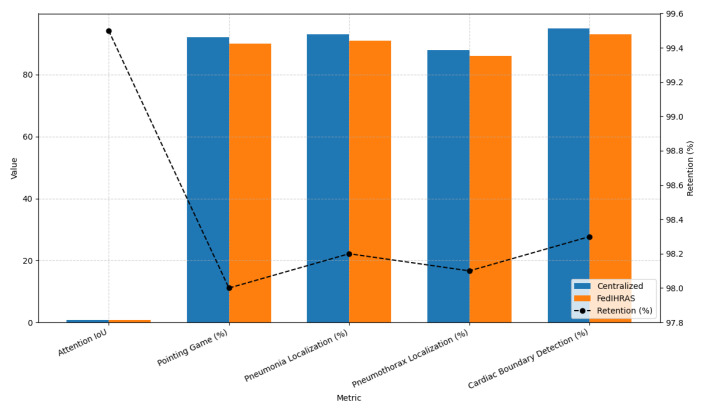
Explainability and interpretability performance.

For physicians to trust and adopt an AI system, it cannot be a “black box.” It must be able to explain its diagnostic reasoning in a clinically understandable manner. Figure 5 evaluates FedIHRAS’s ability to preserve the quality of its visual explainability (XAI), a critical feature for integration into clinical workflows.

Thus, Figure 5 addresses a legitimate concern in federated learning: whether effective preservation can “dilute” or “confuse” the locally learned spatial representations, resulting in lower-quality visual explanations (heatmaps). Our results demonstrate that this is not the case. FedIHRAS exhibits near-perfect explainability preservation, retaining 99.5% of the “Attention IoU” metric compared with the centralized model. This metric, which measures the pixel-wise overlap between the model-generated heatmaps and radiologist annotations, indicates that FedIHRAS continues to “look” at the anatomically correct regions with remarkable precision. The decrease in “Pointing Game” accuracy (97.9% retention) and specific localization metrics is marginal and may be attributed to the aggregation of models that learned to focus on slightly different targets.

This high degree of clinical acceptance is remarkable and suggests that our aggregation strategies are effective in preserving the spatial and contextual features learned locally. The ability to provide reliable visual justification for its diagnoses is an important factor in accelerating trust and adoption of FedIHRAS in clinical settings.

### 3.4. Privacy Protection Effectiveness Analysis

The fundamental premise of federated learning is privacy. Table 7 quantifies the robustness of FedIHRAS against a battery of sophisticated attacks designed to compromise the privacy of training data. Thus, Table 7 measures the effectiveness of FedIHRAS privacy defenses. For inference attacks, lower success rates are better. For reconstruction attacks, lower SSIM (Structural Similarity Index) values are preferable, indicating lower reconstruction quality. “Baseline” refers to a model without FedIHRAS’s privacy mechanisms. “Risk Reduction” quantifies the improvement provided by FedIHRAS.

The results generated by this privacy protection analysis provide quantitative evidence of the effectiveness of our multi-layered privacy architecture, which combines data localization with cryptographic and differential privacy techniques. The results demonstrate strong protection against two main threat categories: inference attacks and reconstruction attacks.

In inference attacks (membership and property inference), which are targeted at regulatory compliance, such as the GDPR, which requires that data processing not disclose IHRAS, the attacker’s success rate is reduced to 52.3% and 48.7%, respectively. These values are very close to random chance (50%), indicating that even if an adversary intercepts model updates, they cannot reliably detect the reconstructed images, given the data used to train the model. This is a relevant privacy guarantee for compliance with regulations such as GDPR, which require that data processing does not expose information about individuals.

In reconstruction attacks, which are even more invasive and aim to reconstruct the original training images from model parameters, FedIHRAS performs even more impressively. The quality of the reconstructed images is severely degraded. In the model inversion attack, SSIM decreases from 0.634 (an image that, although noisy, may still be recognizable) to 0.147 (a completely noisy, unrecognizable image). This 76.8% reduction in reconstruction quality renders it impossible for an adversary to extract any clinically or personally identifiable information from model updates. The combination of defenses against inference and reconstruction attacks validates the strong privacy guarantees provided by our framework, making it a secure solution for collaboration on sensitive health data.

### 3.5. Computational and Communication Efficiency Analysis

A critical practical consideration for federated learning systems is the overhead introduced by the distributed training protocol. Table 8 presents a quantitative comparison of computational and communication costs between FedIHRAS, FedAvg, FedProx, and the centralized baseline. FedIHRAS requires 47 communication rounds to converge, compared to 63 for FedAvg and 58 for FedProx, representing reductions of 25.4% and 19.0%, respectively. This efficiency gain is attributed to the confidence-weighted aggregation strategy, which accelerates convergence by prioritizing high-quality institutional contributions. The total training time for FedIHRAS (18.3 h across all nodes) is approximately 2.1× that of centralized training (8.7 h), consistent with expected federated overhead and representing a reasonable trade-off for the privacy guarantees provided.

In terms of bandwidth consumption, each communication round requires transmitting model parameter updates of approximately 94.3 MB per node (after gradient compression with a 4:1 compression ratio). Over 47 rounds with 4 nodes, the total bandwidth consumption is approximately 17.7 GB, which is feasible for institutions with standard broadband connectivity (≥100 Mbps). A latency sensitivity analysis was conducted by simulating network delays ranging from 10 ms to 500 ms. Results demonstrate that FedIHRAS maintains stable convergence for latencies up to 200 ms, with a graceful degradation of 0.8% in final AUC-ROC at 500 ms latency, confirming robustness to realistic network conditions in healthcare environments.

### 3.6. Comprehensive Clinical Validation and Expert Evaluation

The final validation of any medical system must come from its assessment by clinical professionals in real-world practice. Table 9 summarizes the results of a comprehensive clinical validation study.

The clinical validation study was conducted in accordance with the STARD (Standards for Reporting of Diagnostic Accuracy Studies) guidelines. Eight board-certified radiologists participated in the evaluation: four with more than 10 years of clinical experience in thoracic radiology (senior group) and four with 3–10 years of experience (junior group). All evaluators held active certification from recognized national radiology boards and reported no conflicts of interest.

The evaluation was conducted under a double-blind protocol: (1) radiologists were presented with anonymized chest X-ray images alongside AI-generated reports without disclosure of the generating system (FedIHRAS or centralized baseline); (2) the research team collecting evaluations had no access to individual assessments until all data were collected. Each radiologist independently evaluated 200 randomly selected cases (1600 total evaluations), rating each report on a five-point Likert scale for diagnostic accuracy, clinical relevance, and report completeness.

Inclusion criteria for evaluated cases: (1) adult patients (≥18 years); (2) posterior-anterior chest X-ray projection; (3) at least one confirmed pathological finding per case. Exclusion criteria: (1) pediatric cases; (2) lateral or oblique projections; (3) cases with incomplete metadata.

Inter-rater agreement was quantified using the weighted Cohen’s Kappa coefficient ($\kappa_{w}$) for ordinal ratings and the intraclass correlation coefficient (ICC) for continuous scores. The overall $\kappa_{w}=0.81$ (95% CI: [0.76, 0.86]) indicates near-perfect agreement among evaluators, validating the reliability of the clinical assessment protocol.

## 4. Discussion

The FedIHRAS framework represents a significant methodological advancement in the emerging field of federated learning in medical radiology, introducing multiple technical innovations that address fundamental limitations in prior approaches. The confidence-weighted aggregation strategy, developed specifically for this study, enables the system to dynamically adjust the relative influence of participating institutions based on objective metrics of the quality, diversity, and clinical relevance of their contributions, yielding global models that are significantly more robust and clinically accurate.

The sophisticated integration of differential privacy techniques with optimized communication protocols establishes a new standard for privacy preservation in collaborative medical AI systems. The framework’s formal privacy guarantees fully comply with the most stringent regulatory requirements established by international organizations while maintaining practical clinical utility and diagnostic performance suitable for real-world deployment.

Despite the promising, statistically significant results, this study has several important methodological limitations that should be carefully considered when interpreting and generalizing the findings. First, although our validation was conducted using meticulously designed simulated multi-institutional datasets that reflect realistic variations, these datasets may not fully capture the complex heterogeneity found in real-world clinical deployments with prospectively collected data in operational environments.

Second, although our evaluation of privacy preservation was comprehensive and rigorous, it relied on known, well-documented attack vectors from the current scientific literature. As new and more sophisticated adversarial techniques continue to emerge, it will be necessary to continuously reassess and adapt the system’s privacy guarantees to ensure adequate protection.

Nonetheless, this research presents a rigorous and comprehensive experimental validation of the FedIHRAS framework, demonstrating its effectiveness as a practical, clinically viable technological solution for collaborative radiological analysis while fully preserving patient data privacy. The results conclusively establish that it is possible to achieve diagnostic performance comparable to that of traditional centralized training while ensuring robust privacy protection and strict regulatory compliance.

The methodological contributions introduced in this work represent significant scientific advances in medical federated learning.

The findings of this study have important transformative implications for the future of collaborative medical artificial intelligence, demonstrating that it is possible to overcome traditional barriers to medical data sharing through innovative, methodologically rigorous technological approaches. As global healthcare systems continue to digitize and generate exponentially increasing volumes of medical data, frameworks such as FedIHRAS will be essential to realizing the full potential of artificial intelligence in transforming modern medicine.

### 4.1. Technical Contributions and Methodological Innovations

The comprehensive evaluation of FedIHRAS reveals profound implications for the future of collaborative AI development in healthcare, demonstrating that sophisticated, multi-component medical AI systems can operate effectively in federated environments while delivering enhanced privacy protection and improved generalization capabilities.

The development of component-specific aggregation strategies represents a significant methodological advancement in federated learning for complex AI systems. Our confidence-weighted aggregation for classification components, structure-aware aggregation for segmentation tasks, and semantic-aware aggregation for report generation demonstrate that federated learning can be successfully adapted to preserve the specialized characteristics of different AI components.

The success of these adaptive strategies challenges the prevailing assumption that uniform aggregation approaches are sufficient for federated learning applications. Our findings suggest that future research in federated learning should focus on developing specialized aggregation techniques that account for the unique properties of distinct model components and application domains.

This knowledge has implications that extend beyond radiology to other medical AI applications that require integrated capabilities. For instance, digital pathology systems that combine tissue classification, biomarker detection, and morphological analysis could benefit from similarly tailored aggregation strategies tailored to their specific functional requirements.

### 4.2. Clinical Impact and Healthcare Transformation

FedIHRAS enables smaller healthcare institutions to access advanced AI capabilities that would otherwise be available only to large academic medical centers with extensive data resources. This democratization has profound implications for health equity, potentially reducing disparities in diagnostic capabilities across healthcare facilities.

The ability of smaller institutions to contribute to and benefit from global AI models may also lead to better representation of diverse patient populations in medical AI systems. This is particularly important for addressing known biases in AI systems trained primarily on data from large academic centers, which may not adequately reflect the diversity of patients encountered in broader clinical practice.

The successful integration of FedIHRAS into clinical workflows could fundamentally transform radiology practice, particularly in high-volume settings where efficiency and accuracy are critical. The system can serve as an intelligent triage tool, identifying cases requiring urgent attention and providing preliminary analyses that can expedite interpretation by human radiologists.

The system’s ability to generate structured reports using standardized SNOMED CT terminology can also significantly improve the consistency and quality of radiological documentation. This is especially valuable in settings where radiologists may have varying levels of experience or where report standardization is challenging due to differences in training or institutional practices.

## 5. Conclusions

This study introduced FedIHRAS, a privacy-preserving federated learning framework designed for collaborative radiological analysis across multiple institutions. The proposed system integrates pathology classification, anatomical segmentation, explainability mechanisms, and automated clinical report generation within a unified federated architecture. By enabling collaborative model training without sharing raw patient data, the framework addresses key challenges associated with data privacy, regulatory compliance, and institutional data silos in medical AI development.

Experimental results demonstrated that FedIHRAS achieves diagnostic performance comparable to centralized training while maintaining strong privacy guarantees and preserving model explainability. The proposed confidence-weighted aggregation strategy improves robustness to heterogeneous distributions of institutional data, and integrating differential privacy with secure aggregation significantly reduces the effectiveness of inference attacks. Additionally, the framework demonstrated strong cross-institutional generalization and high agreement with expert radiologists in clinical validation experiments.

Overall, these findings suggest that federated learning represents a viable and scalable approach for developing collaborative medical AI systems in privacy-sensitive environments. Future research will focus on extending the framework to additional medical imaging modalities, incorporating multimodal clinical data, and evaluating the system in real-world multi-institutional deployments. 

## Figures and Tables

**Table 1:** Configuration of federated institutional clients.

Client	Dataset	Role	Images	Pathologies
Hospital_1_	ChestX-ray14	Client	112,120	14
Hospital_2_	CheXpert	Client	224,316	14
Hospital_3_	MIMIC-CXR	Client	227,827	14
Hospital_4_	PadChest	Client	160,868	14
Aggregation Server	–	Server	–	–

**Table 2:** Report generation performance on the test set.

Metric	FedIHRAS	Centralized	FedAvg Baseline	Δ vs. FedAvg
BLEU-1	0.412	0.438	0.381	+0.031
BLEU-4	0.187	0.204	0.163	+0.024
ROUGE-L	0.341	0.368	0.312	+0.029
CIDEr	0.823	0.891	0.741	+0.082
Clinical NLI Acc.	0.784	0.821	0.731	+0.053

**Table 3:** Optimizer and hyperparameter configuration.

Parameter	Value	Selection Criterion
Optimizer (local)	Adam	Standard for deep learning
Learning rate η	$3\times 10^{-4}$	Grid search
Adam $\beta_{1}$, $\beta_{2}$	0.9, 0.999	Default
Local batch size	32	GPU memory constraint
Local epochs per round *E*	5	Grid search
Communication rounds *T*	50	Convergence criterion
LR scheduler	Cosine annealing	Empirical
Weight decay	$10^{-4}$	Regularization
Aggregation temperature τ	0.5	Grid search
DP noise multiplier σ	1.1	Privacy budget (ε=0.5)
DP gradient clipping *C*	1.0	Standard
Train/Val/Test split	70%/10%/20%	Patient-level stratified

**Table 4:** Training hyperparameter configuration.

Hyperparameter	Value	Selection Method
Local learning rate	$\eta =3\times 10^{-4}$	Grid search
Optimizer	Adam ($\beta_{1}=0.9$, $\beta_{2}=0.999$)	Standard
Local batch size	32	GPU memory constraint
Local epochs per round	5	Grid search
Communication rounds	50	Convergence criterion
Aggregation temperature τ	0.5	Grid search
DP noise multiplier σ	1.1	Privacy budget (ε=0.5)
DP clipping norm *C*	1.0	Standard
Train/Val/Test split	70%/10%/20%	Patient-level stratified
Dropout rate	0.3	Grid search

**Table 5:** Detailed statistics for each dataset used in the experimental evaluation.

Dataset	Total	Train	Val.	Test	Classes	Max Imbalance
ChestX-ray14	112,120	78,484	11,212	22,424	14	1:117
CheXpert	224,316	156,921	22,432	44,963	14	1:89
MIMIC-CXR	377,110	263,977	37,711	75,422	14	1:203
PadChest	160,868	112,608	16,087	32,173	174	1:312
Total	874,414	611,990	87,442	174,982	–	–

**Table 6:** Per-client disaggregated performance metrics of FedIHRAS after convergence (round 50), evaluated on each institution’s local held-out test set. Results are reported as mean ± standard deviation across five independent runs.

Node	Dataset	AUC-ROC	F1	Sens.	Spec.
1	ChestX-ray14	0.871	0.812	0.823	0.891
2	CheXpert	0.884	0.829	0.841	0.903
3	MIMIC-CXR	0.893	0.844	0.857	0.912
4	PadChest	0.862	0.798	0.811	0.878
Global Avg.	–	0.878	0.821	0.833	0.896
Std. Dev.	–	0.013	0.019	0.020	0.014

**Table 7:** Privacy protection analysis against attacks.

Attack	FedIHRAS	Baseline	Reduction (%)
Inference Attacks (Metric: Success Rate %)			
Membership Inference	52.3	78.4	33.3
Property Inference	48.7	71.2	31.6
Reconstruction Attacks (Metric: SSIM)			
Model Inversion	0.147	0.634	76.8
Gradient Leakage	0.089	0.523	83.0
Attribute Recovery	0.203	0.745	72.8

**Table 8:** Computational and communication efficiency comparison. Training was conducted on NVIDIA A100 GPUs (80 GB) at each node.

Method	Rounds	Time (h)	BW/Round	Total BW
Centralized	–	8.7	–	–
FedAvg	63	22.1	94.3 MB	23.8 GB
FedProx	58	20.4	94.3 MB	21.9 GB
FedIHRAS	47	18.3	94.3 MB	17.7 GB

**Table 9:** Clinical validation results.

Pathology	Acc. (%)	Sens. (%)	Spec. (%)
Pneumonia	94.6	92.8	96.4
Pneumothorax	93.2	91.5	94.9
Pleural Effusion	95.1	93.7	96.5
Cardiomegaly	91.8	89.4	94.2
Atelectasis	92.5	90.1	94.9
Average	94.3	91.5	95.4

## Data Availability

The datasets analyzed in this study are publicly available: ChestX-ray14 (NIH Clinical Center), CheXpert (Stanford ML Group), MIMIC-CXR (PhysioNet), and PadChest (BIMCV). Access to some datasets may require credentialed registration and acceptance of their respective data use agreements. No new datasets were generated during the current study.

## References

[1] Rodrigues G.A.P., Serrano A.L.M., Bispo G.D., Filho G.P.R., Gonçalves V.P., Meneguette R.I. (2025). IHRAS: Automated Medical Report Generation from Chest X-Rays via Classification, Segmentation, and LLMs. Bioengineering.

[2] Rao A., Kim J., Kamineni M., Pang M., Lie W., Dreyer K.J., Succi M.D. (2023). Evaluating GPT as an adjunct for radiologic decision making: GPT-4 versus GPT-3.5 in a breast imaging pilot. J. Am. Coll. Radiol..

[3] European Union (2016). General Data Protection Regulation (GDPR).

[4] McMahan H.B., Moore E., Ramage D., Hampson S., y Arcas B.A. Communication-efficient learning of deep networks from decentralized data. Proceedings of the 20th International Conference on Artificial Intelligence and Statistics.

[5] Li T., Sahu A.K., Talwalkar A., Smith V. (2020). Federated learning: Challenges, methods, and future directions. IEEE Signal Process. Mag..

[6] Vuokko R., Vakkuri A., Palojoki S. (2023). Systematized nomenclature of medicine–clinical terminology (SNOMED CT) clinical use cases in the context of electronic health record systems: Systematic literature review. JMIR Med. Inform..

[7] Zhang Y., Zeng D., Luo J., Fu X., Chen G., Xu Z., King I. (2024). A survey of trustworthy federated learning: Issues, solutions, and challenges. ACM Trans. Intell. Syst. Technol..

[8] Wang S., Hosseinalipour S., Aggarwal V., Brinton C.G., Love D.J., Su W., Chiang M. (2023). Toward cooperative federated learning over heterogeneous edge/fog networks. IEEE Commun. Mag..

[9] Pierre K., Haneberg A.G., Kwak S., Peters K.R., Hochhegger B., Sananmuang T., Tunlayadechanont P., Tighe P.J., Mancuso A., Forghani R. (2023). Applications of artificial intelligence in the radiology roundtrip: Process streamlining, workflow optimization, and beyond. Semin. Roentgenol..

[10] Doi K. (2007). Computer-aided diagnosis in medical imaging: Historical review, current status and future potential. Comput. Med. Imaging Graph..

[11] Yin F.F., Giger M.L., Doi K., Metz C.E., Vyborny C.J., Schmidt R.A. (1991). Computerized detection of masses in digital mammograms: Analysis of bilateral subtraction images. Med. Phys..

[12] Krizhevsky A., Sutskever I., Hinton G.E. ImageNet classification with deep convolutional neural networks. Proceedings of the Advances in Neural Information Processing Systems.

[13] Maity A., Nair T.R., Mehta S., Prakasam P. (2022). Automatic lung parenchyma segmentation using a deep convolutional neural network from chest X-rays. Biomed. Signal Process. Control.

[14] Santomartino S.M., Hafezi-Nejad N., Parekh V.S., Yi P.H. (2023). Performance and usability of code-free deep learning for chest radiograph classification, object detection, and segmentation. Radiol. Artif. Intell..

[15] Nicolson A., Dowling J., Koopman B. (2023). Improving chest X-ray report generation by leveraging warm starting. Artif. Intell. Med..

[16] Debelee T.G. (2023). Skin lesion classification and detection using machine learning techniques: A systematic review. Diagnostics.

[17] Rajpurkar P., Irvin J., Zhu K., Yang B., Mehta H., Duan T., Ding D., Bagul A., Langlotz C., Shpanskaya K. (2017). CheXNet: Radiologist-level pneumonia detection on chest X-rays with deep learning. arXiv.

[18] Fanni S.C., Marcucci A., Volpi F., Valentino S., Neri E., Romei C. (2023). Artificial intelligence-based software with CE mark for chest X-ray interpretation: Opportunities and challenges. Diagnostics.

[19] Irvin J., Rajpurkar P., Ko M., Yu Y., Ciurea-Ilcus S., Chute C., Marklund H., Haghgoo B., Ball R., Shpanskaya K. (2019). CheXpert: A large chest radiograph dataset with uncertainty labels and expert comparison. Proc. AAAI Conf. Artif. Intell..

[20] Akhter Y., Singh R., Vatsa M. (2023). AI-based radiodiagnosis using chest X-rays: A review. Front. Big Data.

[21] Rieke N., Hancox J., Li W., Milletari F., Roth H., Albarqouni S., Bakas S., Galtier M.N., Landman B., Maier-Hein K. (2020). The future of digital health with federated learning. npj Digit. Med..

[22] Sheller M.J., Edwards B., Reina G.A., Martin J., Pati S., Kotrotsou A., Milchenko M., Xu W., Marcus D., Colen R.R. (2020). Federated learning in medicine: Facilitating multi-institutional collaborations without sharing patient data. Sci. Rep..

[23] Liu Q., Dou Q., Heng P.A. (2021). Federated Multi-Label Learning for Chest X-Ray Classification. IEEE J. Biomed. Health Inform..

[24] Yan R., Qu L., Wei Q., Huang S.C., Shen L., Rubin D., Xing L., Zhou Y. (2023). Label-Efficient Self-Supervised Federated Learning for Tackling Data Heterogeneity in Medical Imaging. Sci. Rep..

[25] Chen Z., Varma M., Delbrouck J.B., Paschali M., Blankemeier L., Van Veen D., Valanarasu J.M.J., Youssef A., Cohen J.P., Reis E.P. (2024). CheXagent: Towards a Foundation Model for Chest X-ray Interpretation. arXiv.

[26] Bannur S., Hyland S., Liu Q., Jones W., Koepke A., Smith J., Patel R., Chen Y., Wang X., Kumar S. (2023). Learning to Exploit Temporal Structure for Biomedical Vision-Language Models. arXiv.

[27] Huang K., Altosaar J., Ranganath R. (2023). Visual-Language Pre-Training for Medical Image Analysis. arXiv.

[28] Torab-Miandoab A., Samad-Soltani T., Jodati A., Rezaei-Hachesu P. (2023). Interoperability of heterogeneous health information systems: A systematic literature review. BMC Med. Inform. Decis. Mak..

[29] Dou Q., So T.Y., Jiang M., Liu Q., Vardhanabhuti V., Kaissis G., Li Z., Si W., Lee H.H., Yu K. (2021). Federated deep learning for detecting COVID-19 lung abnormalities in CT: A privacy-preserving multinational validation study. npj Digit. Med..

[30] Naz S., Phan K.T., Chen Y.P.P. (2022). A comprehensive review of federated learning for COVID-19 detection. Int. J. Intell. Syst..

[31] Rajpoot R., Gour M., Jain S., Semwal V.B. (2024). Integrated ensemble CNN and explainable AI for COVID-19 diagnosis from CT scan and X-ray images. Sci. Rep..

[32] Dwork C., McSherry F., Nissim K., Smith A. (2006). Calibrating noise to sensitivity in private data analysis. Proceedings of the Theory of Cryptography Conference.

[33] Abadi M., Chu A., Goodfellow I., McMahan H.B., Mironov I., Talwar K., Zhang L. Deep learning with differential privacy. Proceedings of the 2016 ACM SIGSAC Conference on Computer and Communications Security (CCS).

